# Effect of Diatomite Application on the Removal of Biogenic Pollutants in Rain Gardens

**DOI:** 10.3390/ma17246279

**Published:** 2024-12-22

**Authors:** Agnieszka Grela, Michał Łach, Justyna Pamuła, Karolina Łach, Izabela Godyń, Dagmara Malina, Zbigniew Wzorek, Kinga Setlak, Damian Grela

**Affiliations:** 1 Faculty of Environmental and Power Engineering, Cracow University of Technology, Warszawska 24, 31-155 Cracow, Poland; justyna.pamula@pk.edu.pl (J.P.); karolina.lach@pk.edu.pl (K.Ł.); izabela.godyn@pk.edu.pl (I.G.); 2 Faculty of Material Engineering and Physics, Cracow University of Technology, Jana Pawła II 37, 31-864 Cracow, Poland; michal.lach@pk.edu.pl (M.Ł.); kinga.setlak@pk.edu.pl (K.S.); 3 Faculty of Chemical Engineering and Technology, Cracow University of Technology, Warszawska 24, 31-155 Cracow, Poland; dagmara.malina@pk.edu.pl (D.M.); zbigniew.wzorek@pk.edu.pl (Z.W.); 4 Faculty of Electrical and Computer Engineering, Cracow University of Technology, Warszawska 24, 31-155 Cracow, Poland; damian.grela@pk.edu.pl

**Keywords:** runoff treatment, bioretention, phosphates, nitrates, sorbent

## Abstract

Due to its structure and properties, diatomite from a deposit in Jawornik Ruski (Subcarpathian Voivodeship) can be used as a sorbent in rain gardens. The purpose of the current research is to analyze how enriching the substrate used in a rain garden with diatomite can affect the removal of biogenic pollutants. This study was carried out under laboratory conditions using retention columns, two experimental columns with different contents of diatomite, and a control column without the addition of diatomite. Analyses of the materials used included studies of the characteristics of the rain garden layers (water permeability and granulometric analysis) and characterization of the diatomite (SEM images, oxide and phase composition, leachability, and BET). The effects of diatomite on pollutant removal were studied for NH_4_^+^, PO_4_^3−^, NO_3_^−^. The results showed approximately 3-fold higher reductions in the concentration of NH_4_^+^ and PO_4_^3−^ in the columns with the addition of diatomite than in the control one (reduction in the concentration of NH_4_^+^ by 93 and 94% and of PO_4_^3−^ by 94 and 98% with the addition of 20 and 30% diatomite contents, respectively). The study results confirmed the possibility of removing contaminants using diatomite, thus reducing their entry into the aquatic environment.

## 1. Introduction

Rainwater in cities has recently become the focus of many researchers. Accelerated urbanization is bringing increased attention to the volume of rainwater runoff and the potential for rainwater retention [1,2]. A key reason for this interest is the desire to both control and reduce the pollution contained in rainwater. One solution that is easy to apply with respect to the existing infrastructure of cities is a so-called rain garden [3,4]. This solution is often referred to as a biofilter and is categorized as a low-impact development (LID) method. Rain gardens play an important role in the development and management of rainwater, and they contribute to improvements in water quality. This change arises because in a rain garden, due to the filtration process, the pollutants that are washed off impermeable surfaces can be effectively retained [5]. The literature has described options for the removal of many contaminants [6], with the main problems involving the effective removal of various forms of nitrogen [7,8] and phosphorus [9]. Biogenic compounds, otherwise known as nutrients, can cause eutrophication in bodies of water that are recipients of rainwater, such as water reservoirs [9]. It is well-known that rain gardens can remove nitrogen (N) through microbial processes, including the mineralization of organic N to NH_4_^+^, the nitrification of NH_4_^+^ to NO_2_ by ammonia oxidizing bacteria (AOB) and then to NO_3_ by nitrite oxidizing bacteria (NOB), and the denitrification of NO_2_/NO_3_ to N_2_. It is known that nitric oxide (N_2_O)—a typical climate gas—is released during the nitrogen removal process in rain gardens. Nitric oxide is a byproduct of nitrification and an intermediate product of denitrification [10]. Wang H. et al. [10] studied the nitrogen removal processes (nitrification and denitrification) in rain gardens by comparing a conventional rapid-filtration rain garden and a modified rain garden with an internal water storage system, describing the removal processes with the consideration of the bacterial community composition. They proved that ammonium (NH_4_^+^) can be effectively removed by negatively charged soil particles and can then be separated from rain garden leachates, while NO_2_/NO_3_ tends to be released during discharge. In addition, the study by Wang S. et al. [11], which included an observation spanning one year of operation of 18 stepped planted bioretention systems, showed the occurrence of nitrification and denitrification processes in them, and the influence of the choice of plant species was also determined. Peterson focused on the study of denitrification processes by examining bioretention cells containing an internal water storage zone (with organic carbon substrate derived from wood chips) and showed that nitrogen (N) can be removed completely from rainwater using denitrification and conversion to N_2_, which can then be released into the atmosphere [12]. The denitrification process can be controlled in a rain garden by providing a permanent anaerobic environment. This happens, for example, when there is an increase in the outlet of the rain garden. Morse et al. [13] also studied denitrification processes in stormwater basins (structures analogous to rain gardens) and showed that an internal water storage system with the addition of sustained-release carbon, which can be derived from various sources, increases the efficiency of denitrification [13]. Jang et al. [14] presented the total nitrogen removal performance characteristics of bioswale and rain garden systems from 2014 to 2017, showing a median value of pollutant removal in rain garden of 54.32% (lower than in bioswales: 74–81%). Gilchrist et al. [14] studied the operation of eight outdoor rain gardens without vegetation to evaluate the effects of hydraulic loading, the presence/absence of a layer of buried wood chips, and the presence/absence of a subsurface saturated zone (SSZ) on nitrate and nitrite removal. General conclusions from their study highlighted the importance of incorporating a SSZ for nitrate and nitrite load reduction and demonstrated that a carbon source did not make a significant contribution to N removal. Despite the considerable amount of research on nitrogen removal in rain gardens, research into the mechanisms of nitrogen removal in rain gardens is at an early stage [15].

Phosphorus compounds have the greatest impact on the eutrophication of surface waters among the pollutants found in rainwater [16,17,18,19]. Phosphorus in stormwater runoff occurs in the forms of particulate phosphorus (PP) and dissolved phosphorus (DP). Reductions in phosphorus concentrations in filter media are achieved by retaining phosphorus through physical and biogeochemical processes [20,21]. In the case of DP, two forms are distinguished: soluble reactive phosphorus (SRP) and dissolved organic phosphorus (DOP). In the case of both of these forms of DP, the removal mechanisms include plant uptake, microbial uptake, and sorption [21]. The most commonly used method for removing phosphorus compounds from water is chemical precipitation with metal salts, during which the phosphorus sorption process (this process mainly concerns the fraction of inorganic dissolved phosphorus as organic phosphorus does not participate in this process) takes place as clay minerals participate in the process. Various natural (clays, clayey sands, marls, limestones, and gypsums) or artificial materials are used as fillings for sorption column beds [18,19,22]. It has been shown that the efficiency of phosphate removal depends mainly on the contents of calcium, aluminum, and iron compounds in the bed [21]. Another effective and commonly used method for removing phosphorus compounds from water (e.g., from sewage) is based on the use of specific activated sludge bacteria, which are capable of accumulating phosphorus in their biomass in greater quantities than their metabolic demand. During biological treatment, organic phosphorus is oxidized to phosphates [22,23,24]. Phosphate removal in iron-enhanced rain gardens was studied by Weiss et al. [25] and showed that the SRP retention in the iron-enhanced rain garden was initially almost 98%, but it gradually decreased to about 91% after more than 61 m of water had infiltrated. An earlier study of iron addition to sand filters used for 1 year for stormwater treatment was also reported by Erickson et al. [26]. In this study, 88% phosphate capture was demonstrated. Ament et al. [27] studied the effect of adding drinking water treatment residuals (containing aluminum and iron) to bioretention soil media on phosphorus removal and showed near 100% removal. Søberg et al. [28] investigated the effect of a submerged zone with embedded carbon source, temperature, and (road) salt on phosphorus removal in a planted bioretention column with a sand-based filter material. A DP removal of 84% was demonstrated. It is also worth emphasizing that some scholars, such as Kohlsmith et al. [29], showed 41% increases in phosphate concentrations in the outflow of established lined bioretention facilities in Portland, Oregon, and a review study [30] conducted on data from the International Stormwater Best Management Practices (BMP) Database showed that a number of existing facilities in the US increase phosphate concentrations in the outflow.

Due to the fact that the processes of removing biogenic pollutants in rain gardens and whether the removal of formed nitrogen results in higher emissions still have not been clearly determined, there is a need to conduct research, such as that proposed by the authors of this paper. The study of processes occurring in conventional rain gardens and in rain gardens with the addition of sorbents allows for a better understanding of the processes of nutrient removal in rain gardens.

Sorbents, both natural and synthetic, are popular materials used to remove nutrient pollution from aquatic environments [31,32]. Natural sorbents include diatomites (siliceous sedimentary rocks), which are formed from the accumulation of microscopic diatom shells (i.e., single-cell algae). Diatom shells consist mainly of SiO_2_ and have an extensive structure, which gives diatomites high porosity and the ability to absorb various substances, contributing to their possession of a number of specific properties, which are useful in various applications. At present, the most common applications of diatomite are as a sorbent for petroleum substances [33,34], in cleaning materials and cosmetics, in animal breeding [35], and in agriculture or horticulture [36]. Diatomites are capable of controlling insects or parasites and are used as a feed additive or soil conditioner. Diatomites are often appreciated in organic farming due to their ability to retain moisture and stimulate the development of the root systems of plants while maintaining their natural character, providing an alternative to pesticides.

Diatomites can also be used as sorbents for heavy metals and other contaminants in aquatic environments [37,38,39]. However, diatomite or diatomaceous earth is rarely used to remove nitrate, ammonium, and phosphate. Nevertheless, there are literature reports confirming the effectiveness of diatomite use in this context. One study [40] investigated the impact of the size of the grain fraction of diatomite and the effects of modification methods on the maximum sorption capacity of diatomite, as well as the cation exchange capacity against PO_4_^3−^, NO_3_^−^, and SO_4_^2−^ ions. The experiments carried out showed that diatomite effectively stabilizes the absorbed ions on the inner surface and can be used as a filter to prevent agricultural pollution and river eutrophication. Diatomites have also been investigated for their use as filter media in water treatments, wastewater treatments, and the permeable reactive barriers (PRBs) of aquifers. In the case of beds used to remove nitrogen compounds, diatomite is successfully used as a component of biosorption beds to remove ammonium ions. It has been proven [22] that diatomite-based beds accelerate the development of the nitrification bed because, due to their complex chemical composition, they are good carriers and habitats for nitrifying bacteria, which are essential in the process of oxidation of N-NH_4_^+^ to N-NO_3_^−^. Xie et al. [41] conducted a study of PO_4_^3−^ sorption using modified diatomite. Among other things, they showed that phosphate sorption on raw diatomite is strongly dependent on the pH. Diatomite modification (calcining) caused the surface of the adsorbent to be positively charged, which is favorable for the adsorption of the PO_4_^3−^ anion; although the sorption capacity still decreases with increasing pH (the range of 5–10 was studied), its values are over 40 times higher. Wajima et al. [42] prepared zeolite from paper sludge ash with added diatomite and studied its application for the removal of NH_4_^+^ and PO_4_^3−^. They attributed the NH_4_^+^ removal process to cation exchange. In turn, they explained the mechanism of PO_4_^3−^ removal by the formation of insoluble calcium-phosphate salts (zeolites showed released (soluble) Ca^2+^). Zhou et al. [43] studied the application of diatomite as a natural material in permeable reactive barriers (PRBs) for NH_4_^+^ removal in aquifers. Diatomite was enriched with the bacterial powder of nitrifying bacteria. The reduction in NH_4_^+^ from 45 and 55 mg/L to 20 mg/L was achieved. The kinetics of NH_4_^+^ adsorption and the influence of the presence of other cations (Na^+^, Ca^2+^, Mg^2+^ and K^+^) on the adsorption capacity were studied, and it was shown that their presence had little influence on the NH_4_^+^ adsorption capacity because ammonium cation was preferentially absorbed.

The aim of this study was to compare the efficiency of nutrient removal with diatomite, especially NO_3_^−^, NH_4_^+^, and PO_4_^3−^. In addition, thanks to the use of the sorbent in the form of diatomite (in different ratios), it was possible to determine the potential for using this type of material in rain gardens. The sorbent was thoroughly characterized in order to better understand its potential impact on the efficiency of nutrient removal in rain gardens. The results presented in this paper confirmed the suitability of designing a rain garden in a container with diatomite as a sorbent. In the experiment, rainwater under controlled conditions, lacking other micropollutants, was introduced into three model columns differing in filling to accurately assess the removal efficiencies of nitrate (NO_3_^−^), ammonium ions (NH_4_⁺) and phosphate (PO_4_^3−^). The originality of the study consists of the use of diatomite as a sorbent material for biogenic pollutants occurring in urban stormwater runoff. To the authors’ knowledge, this material is not commonly used in rain gardens. Diatomite, being natural, safe, cheap, and commercially available, can easily be used in individual rain gardens. Its use can significantly improve the efficiency of such structures, helping to reduce surface water pollution in urban areas.

## 2. Experiments

### 2.1. Materials for the Construction of Columns—Rain Gardens

The selection of materials was guided by recommendations provided by Climate–Energy–Water Management (pol. Klimat-Energia-Gospodarka Wodna, KEGW), the first specialized local government entity established for the adaptation of cities to climate change. KEGW, in its vademecum for residents, recommends that a rain garden should be built in a sealed container filled with suitable permeable materials [44]. In the experiment conducted, the KEGW orders were followed (Column 1), and part of the filter material layer (sand) was replaced with sorbent (in the form of diatomite) in two different proportions (Column 2 and Column 3). The following materials were used to fill the columns: dolomite grit (hereinafter referred to as dolomite), quartz sand (hereinafter referred to as sand), and decorative gravel (hereinafter referred to as gravel), which were purchased from a local store, as well as diatomite, which came from the diatomite mine in Jawornik Ruski (Podkarpackie Voivodeship, Poland). The supplier of the material was the mine’s owner, Specialized Mining Company Górtech Ltd. Raw diatomite was prepared by a mechanical process consisting of grinding in a rotary ball mill for 5 min and then separation. The material was properly prepared for testing by separating the appropriate fractions, using a Fritzsch Pulverisette 13 laboratory sifter with a suitable set of sieves for this purpose.

The magnitude of the filtration coefficient corresponding to a water temperature of 10 °C (k_10_) was determined for each column filler material according to Polish Standard PKN-CEN ISO/TS 17892-11 [45]. In laboratory tests, the constant gradient method and Darcy’s low for laminar flow were used. The test was conducted using ZWK II apparatus (ZAN Kraków). The porosity n (the ratio of pore volume to total volume) was determined according to the Polish Standards PKN-CEN ISO/TS 17892-1, 17892-2, and 17892-3 by measuring bulk density, water content, dry density, and particle density [46,47,48]. The particle density was determined using gas pycnometer Pycnomatic ATC (Thermo Fisher Scientific, Massachusetts, MA, USA) for diatomite, and for the other materials, it was determined using a fluid pycnometer. Detailed information is included in Supplementary Materials.

The determination of k_10_ for the different materials was important to determine the filtration velocity of the simulated rain. The use of soils with similar permeability in all three columns minimized the experimental error associated with potential different water velocities. Moreover, the amounts of diatomite addition in C2 and C3 were selected on the basis of the k_10_ value. The addition of diatomite (with very good permeability) to sand (with good permeability) in weight ratios of 1:4 and 1:2 allowed for these mixtures (C2 and C3) to have good permeability. This ensured that the water flow in all three columns was at similar velocities and that the filtration conditions recommended by KEGW were maintained.

The results are shown in Table 1.

The granulometric compositions of the materials used were also examined. The tests were carried out according to the technical specification PKN-CEN ISO/TS 17892-4 [50] using the sieve method (laboratory sifter Mulitserw, Morek Company). On the basis of the grain size curves, the contents of individual fractions, d_10_ (effective size), C_u_ (coefficient of uniformity), and C_c_ (coefficient of curvature) were determined for the individual materials according to PN-EN ISO 14688-1 [51] and PN-EN ISO 14688-2 [52]. The results obtained are presented in Table 2.

### 2.2. Analytical Methods

Oxide XRF analysis was performed using a Shimadzu EDX-7200 (Shimadzu Europa GmbH, Duisburg, Germany). The mineralogical composition of diatomite was investigated by means of an X-ray diffraction technique using a PANalytical Aeris (Malvern Panalytical, Almelo, Netherlands) diffractometer. X-ray diffractometry (XRD) is a technique that uses X-ray diffraction to determine the crystalline phases that comprise the object under study. Quantitative analysis was performed using the Rietveld method, which was implemented in the HighScore Plus software. Diffractograms were recorded using Cu-Kα radiation in the scan range of 10–100° with a step of 0.003° (2θ) and a time per step of 340 s using Cu Kα radiation.

The specific surface areas and porosities of the samples were determined using a Quantachrome Autosorb iQ-MP physical sorption analyzer. Volumetric measurements of nitrogen adsorption and desorption were carried out at relative pressures in the range from 1∙10^−6^ to 0.995. Measuring cells with an inner diameter of ø9 mm were used.

The degassing process was carried out in several stages. The set parameters of the degassing process are presented in Table 3. The results were analyzed using the ASiQwin program. The tests of the specific surface area, pore volume, and pore diameter were carried out using a Quantachrome Autosorb iQ-MP physical sorption analyzer (Anton Paar, Graz, Austria). The sample degassing process was carried out in several stages:

1. Heating to the degassing temperature of 80 °C at a rate of 2 °C min^−1^ with a soaking time of 30 min.
2. Heating to 120 °C at a rate of 2 °C min^−1^ and soaking for 30 min.
3. Heating to the degassing temperature of 350 °C at a rate of 5°C min^−1^ with a soaking time of 300 min.

The degassing process should be carried out primarily to remove any remaining moisture adsorbed on the surface of the sample, and it should also ensure the elimination of other gaseous and non-gaseous contaminants. The degassing process enabled an accurate determination of the available pore surface area of the material, which is crucial for calculating specific surface area, pore volume, and pore size distribution.

The specific surface area was determined using the single-point and multi-point Brunauer–Emmett–Teller (BET) method. The pore volume and pore diameter were determined using the Barrett–Joyner–Halenda (BJH) method. The microporosities of the samples were determined using the Dubinin–Raduszkiewicz (DR) method. The results were analyzed with the Quantachrome ASiQwin 5.2 software.

Images of the morphologies of the samples of all diatomite variants were taken using scanning electron microscopy (SEM) using a JEOL IT 2000 microscope (JEOL, Akishima, Tokyo, Japan) for the study. To carry out the observations with the scanning electron microscope, each material was attached using a special carbon adhesive and carbon disks. Both the adhesive and the disks were used to better attach the material, leading to the better conduction of the material. The samples were placed on metal tables and then in a holder and, immediately prior to testing, the surface of the material was coated with a conductive gold layer using a DII-29030SCTR Smart Coater vacuum sputtering machine (Jeol Ltd., Peabody, MA, USA).

Analyses of NH_4_^+^, NO_3_^−^, and PO_4_^3−^ concentrations in water samples were carried out using a microprocessor photometer for water and wastewater analyses (type LF300) from Slandi (Michalowice, Poland). The following reagents were used: for NH_4_^+^, ammonium K (range of determination 0.05–5.0 mg L^−1^), for NO_3_^−^, nitrate K (range of determination 0.1–50.0 mg L^−1^), and for PO_4_^3-^, phosphates LR K (range of determination 0.1–3.5 mg L^−1^), which were also obtained from Slandi (Michalowice, Poland). Prior to the determinations, samples were filtered using qualitative medium filters made of pure cellulose and cotton fluff (type: 390; weight: 84 g/m^2^) purchased from Krakchemia (Krakow, Poland).

A CX-505 laboratory multifunction instrument from ELMETRON (Zabrze, Poland) was used to measure the pH, electrolytic conductivity (EC), salinity (in terms of NaCl and KCl), total dissolved solids (TDS), and dissolved oxygen concentration (O_2_). The technical characteristics of the CX-505 are shown in Table 4.

### 2.3. Columns—Rain Gardens

**Figure 1:**
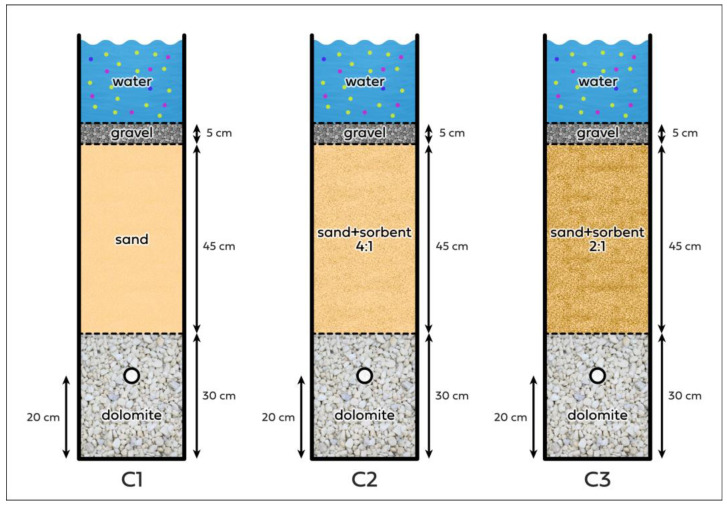
Diagram of the structures of the columns.

Three filter columns (Figure 1) were constructed from epoxy-coated plexiglass pipes purchased from Erson (Kuźnia Raciborska, Poland). The dimensions of the pipe were an outer diameter of 150 mm, inner diameter of 140 mm, and height of 1000 mm. From the bottom, the pipe was blinded with a 150 mm/140 mm plexiglass profiled cover from Erson (Kuźnia Raciborska, Poland), creating a sealed column. According to the manufacturer, the used pipes were resistant to mechanical damage, adverse atmospheric conditions, and UV radiation and did not react with acids, alkalis, or salts [53]. The filter columns served as a laboratory reproduction of a rain garden in a container according to the KEGW recommendations [44]. According to the guidelines, an HDPE tap with a 3/4″ thread from ALCHEM (Torun, Poland) was located in each of the columns at a height of 200 mm from the bottom, which provided a place for water to flow out of the column.

The columns were filled with filter materials. The fill of column 1 (C1) was prepared according to KEWG recommendations. Diatomite, sand, and gravel with weights of 7.02 kg, 11.50 kg, and 1.30 kg, respectively, were used, piling up layers with thicknesses of 30 cm, 45 cm, and 5 cm. In column 2 (C2) and column 3 (C3), the fill was modified by adding diatomite sorbent to the sand layer in varying amounts while maintaining a thickness of 45 cm. For C2, the sand layer with diatomite was 9.44 kg mixed in a weight ratio of 4:1 (sand:diatomite). For C3, the layer of sand and diatomite was 8.85 kg, and it was prepared in a weight ratio of 2:1 (sand:diatomite).

### 2.4. Synthetic Stormwater Runoff

Recent research on environmental pollution control has shown that stormwater runoff is an important indicator for the determination and characterization of pollutants in numerous ecosystems [54]. As pollution is present in the air, all forms of atmospheric precipitation act as an efficient pathway for the removal of gases and diverse particles present in the atmosphere as a consequence of natural and anthropogenic airborne pollutant transport and climatic conditions [55,56]. However, much stronger water pollution occurs in surface runoff, and the type of pollution and its concentration depend largely on the type of land use [57,58].

This study focused on assessing the efficiencies of removing three biogenic substances (NH_4_^+^, NO_3_^−^, and PO_4_^3−^ ions), which are commonly present in stormwater runoff. The assumed concentrations of biogenic components in synthetic stormwater runoff were assumed based on the following:

1. Reported concentrations of forms of nitrogen and phosphorus in rainwater by the Chief Inspectorate of Environmental Protection (pol. Główny Inspektorat Ochrony Środowiska, GIOS) [59].
2. Concentrations at facility inlets collected in the International Stormwater Best Management Practices Database [60].

Measurement results of precipitation chemistry in Poland from 2018 to 2023 showed the following maximum concentrations: nitrogen in the forms of nitrate and nitrite (N-NO_3_^−^ and NO_2_^−^), 2.99 mg L^−1^ (measured in Poznań in 2018); ammonium nitrogen (N-NH_4_^+^), 3.78 mg L^−1^ (measured in Kalisz in 2018); and general phosphorus, 0.581 mg L^−1^ (measured in Poznań in 2022). The BMP DBase, on the other hand, reports the following maximum and median concentrations per inlet to the facilities: N-NO_3_^−^, max 28.0 mg L^−1^, median 0.44 mg L^−1^; N-NH_4_^+^, max 2.192 mg L^−1^, median 0.29 mg L^−1^; and PO_4_^3−^, max 6.98 mg L^−1^, median 1.37 mg L^−1^. For the preparation of the synthetic stormwater runoff solution, salts (NH_4_Cl, KNO_3_, and Na_2_HPO_4_) were used, and the aforementioned reported concentration values were recalculated into concentrations of the individual ions NO_3_^−^, NH_4_^+^, and PO_4_^3−^ (Table 5).

The median concentrations reported in the BMPDB, in the case of NO_3_^−^ and NH_4_^+^, reached values far below the concentrations reported in analyses of rainwater chemistry in Poland, which are likely to be further polluted after stormwater runoff; only PO_4_^3−^ presented similar values. In the end, it was decided to adopt concentration values close to the data reported by GIOS. The reported concentrations were rounded up to the following values: NO_3_^−^ = 15.0 mg L^−1^, NH_4_^+^ = 5.0 mg L^−1^, and PO_4_^3−^ = 2.0 mg L^−1^.

The synthetic stormwater runoff with the composition reported in Table 6 was prepared by dissolving appropriate amounts of analytical grade salts (NH_4_Cl, KNO_3_, and Na_2_HPO_4_; purchased from the WARCHEM Sp. z o.o. company) as sources of the tested biogenic ions in deionized water.

### 2.5. Rain Amount and Duration

The amount of stormwater flowing into the rain garden was determined by the size of the drainage area from which stormwater flowed into the garden and by the intensity of the rainfall. The required size of a rain garden, according to KEGW recommendations, is a minimum of 2% of the drainage area [44], while other Polish cities recommend building a garden with a size of 3–6% of the drainage area [61,62]. Due to the increasing frequency and intensity of intense rainfall in Krakow, it was assumed that the garden would be sized at 6% of the drainage area. The columns had a diameter of 15 cm and an area of 176.71 cm^2^, which allowed for the drainage of a catchment area of 0.2945 m^2^.

In accordance with the KEGW recommendations, the experiment simulated a rainfall supply with a frequency of occurrence of c = 10 years [63]. Precipitation with a 30 min duration was assumed, and a hyetograph of precipitation was adopted according to the precipitation model developed for Krakow, which provided an amount of precipitation in 5-min intervals (Table 7). The total amount of precipitation during 30 min was 31.56 mm [64].

An estimation of the amount of stormwater from the drainage area (0.2945 m^2^) during the model rainfall is shown in Table 7.

### 2.6. Experiment of Sorption of Biogenic Compounds in Columns

**Figure 2:**
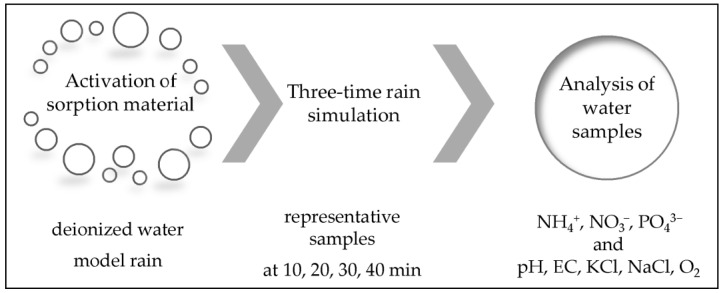
Schematic diagram of the experiment.

The performed experiment included three basic steps (activation of the sorption material, rain simulation, and analysis of water samples), which are detailed in Figure 2.

The material filling the columns was free-flowing and non-hydrated. All three columns were activated. Activation consisted of slowly filling each column with water so that the sorption material was fully saturated. As part of the activation, the columns were flooded twice with deionized water (4.5 l and 2 l) and rinsed with model rain (5 l). The water was poured in slowly to ensure that the column fill structure was not disturbed and to hydrate as many pores as possible. Model rain was also applied during activation to obtain the conditions in which the columns would operate. The duration of activation was 24 h for each of the stages.

After activation of the columns, rainfall simulations were performed. Three such simulations were carried out with amounts and durations according to the assumptions described above. For each column, leachate was collected and representative samples with a volume of 100 mL was taken at 10, 20, 30, and 40 min after the start of the experiment. A sample of model rain was also taken at each sampling time in order to control its quality. In all collected samples, the concentrations of the individual ions NO_3_^−^, NH_4_^+^, and PO_4_^3−^, as well as other parameters, were determined according to Table 4.

## 3. Results

### 3.1. Characteristics of Diatomite

**Figure 3:**
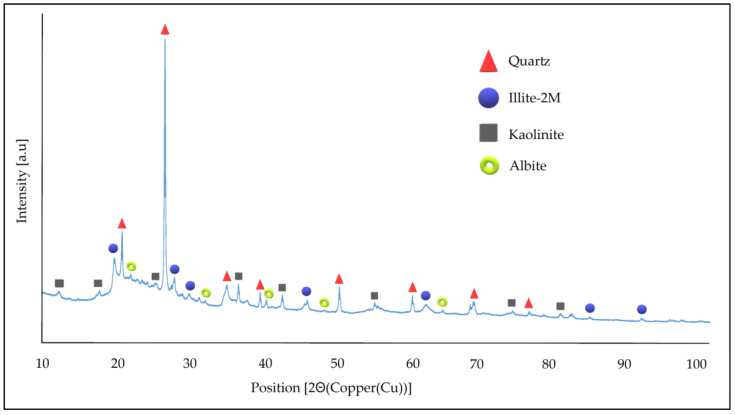
X-ray diffraction pattern for raw diatomite powder.

Table 8 shows the oxide composition of the diatomite used in the experiments, while Figure 3 shows the XRD mineralogical analysis. The test was carried out in an air atmosphere with holders designed for bulk materials and Mylar film, and the results are shown in Table 9. Only the most important oxides detected in the chemical composition, with weight share higher than 0.1, are shown in the table. The composition of diatomite is dominated by silica and Al_2_O_3_. This material is specific due to the fact that diatomites are usually characterized by a SiO_2_ content of more than 80% by weight. Table 9 shows the quantitative phase composition determined via the Rietveld method. The occurrence of phases such as quartz, illite, kaolinite, and albite has been identified in diatomite occurring in Poland by other authors [65], and similar phases have been identified for other types of diatomite by Jindi and his team [66].

The X-ray diffraction pattern of raw diatomite is presented in Figure 3.

**Figure 4:**
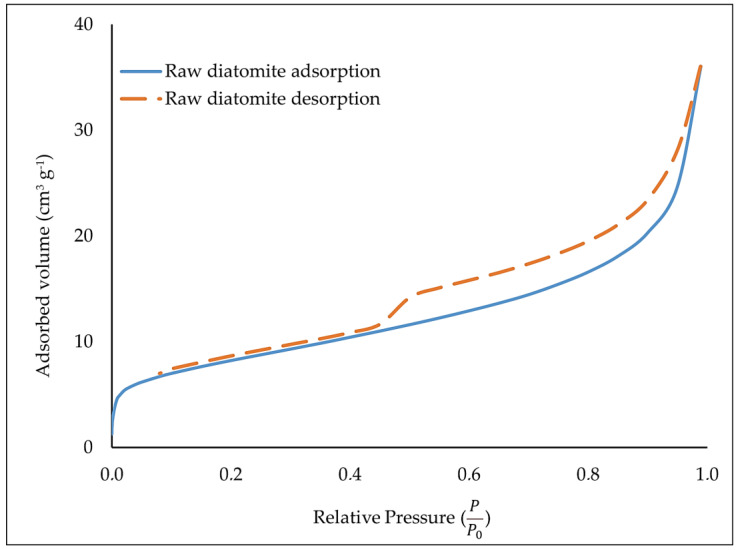
Nitrogen adsorption and desorption isotherms for diatomite.

Figure 4 shows the nitrogen adsorption–desorption isotherms of raw diatomite.

The nitrogen adsorption–desorption isotherms presented above, according to the IUPAC classification, can be described as type IV isotherms. A characteristic H3-type hysteresis loop can be seen for the obtained isotherms.

Table 10 shows porous texture parameters of diatomite. The specific surface area of S_BET_ raw diatomite was 29.003 m^2^ g^−1^, and the volume of micropores in this material oscillated around 0.012 cm^3^ g^−1^. In contrast, the volume of mesopores in crude diatomite was 0.053 cm^3^ g^−1^, and the average pore diameter oscillated around 7.7 nm.

SBET—specific surface according to Brunauer–Emmett–Teller (BET) method, m^2^ g^−1^.

$V_{tot}^{0.99}$—total specific volume of pores for a relative pressure p/p_0_ = 0.99, cm^3^ g^−1^.

$V_{mik}^{DR}$—volume of micropores (pores with widths under 2 nm) according to the Dubinin–Radushkevich method, cm^3^ g^−1^.

$V_{mez}^{BJH}$—volume of mesopores (pores with a width greater than 2 nm and less than 50 nm) according to the Barrett–Joyner–Halve (BJH) method, cm^3^ g^−1^.

$S_{mik}^{DR}$—surface of micropores (pores less than 2 nm wide) according to the Dubinin–Radushkevich method, m^2^ g^−1^.

$S_{mez}^{BJH}$—surface of mesopores (pores with a width greater than 2 nm and less than 50 nm) according to the Barrett–Joyner–Halve (BJH) method, m^2^ g^−1^.

D_A_—average pore diameter, nm.

**Figure 5:**
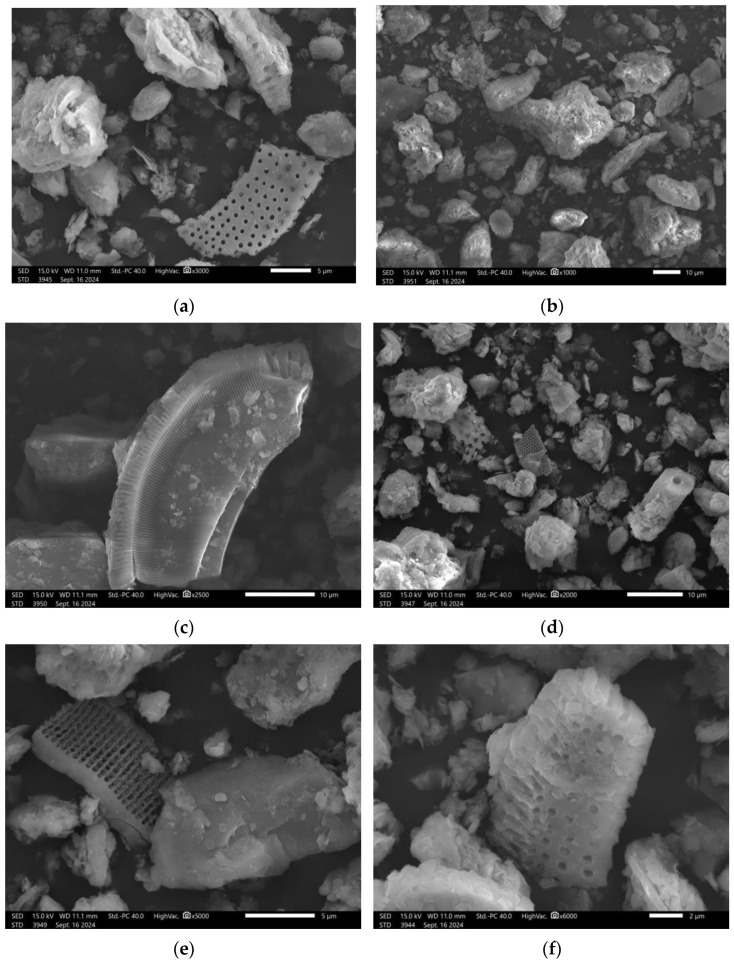
SEM images of diatomite at magnifications: (**a**) 3000×; (**b**) 2500×; (**c**) 5000×; (**d**) 1000×; (**e**) 2000×; and (**f**) 6000×.

The microphotographs (Figure 5) show diatom shells occurring in the materials, taking a variety of forms, which is characteristic for diatomite and diatomaceous earth. The raw material used in the study is a specific material occurring locally, which differs from other diatomites from other beds. The peculiarity of the Polish diatomite is that it is not a pure material and does not contain large amounts of diatomite residues. As shown in the microphotographs above, diatom carapaces are visible, but most of the material occurs in other forms as irregular particles without cellular structures.

The shape of diatoms’ carapaces can be really varied from flat structures (Figure 5a,d) to spatially geometric forms (Figure 5b,c,f) to cylindrical forms (5e). A common feature of all diatom carapaces is the presence of characteristic pores on their surface. In addition, from Figure 5, we can see the size distributions of diatomite structures, which oscillate between 5 µm and even 50 µm.

### 3.2. Results from Experiments

#### 3.2.1. Parameter Changes (pH, PEV) for C1, C2, and C3 in Three Rainfalls

Electrical conductivity is a basic parameter for the initial assessment of water quality. Its value depends on the concentrations of dissolved substances in water, and its changes indicate a change in the physicochemical state of water [67]. The EC value, defined in μS cm^−1^, approximately corresponds to the mineralization of water (in mg L^−1^ [68].

During the three rainfall simulation experiments, regardless of the column fill, an increase in EC relative to the model rain was observed (Table 11). During the first rainfall, the highest increase of average EC relative to the model rain was observed for C3 (81%), and the lowest was observed for C1 (49%). This indicates intensive leaching of contaminants from the column fill material. As the addition of diatomite increased, the amount of solutes increased. On the other hand, during the third rainfall simulation, pollutants were still released from column 1 (an increase in EC of 57% relative to the model rain) while, in columns 2 and 3, the increase in EC relative to the model rain was lower (33% and 32%, respectively). The amount of substances leached from the C1 fill material was almost constant during the three rainfall simulations. A comparison of EC values between the third and first experiments indicated a 5% increase in the value of this parameter. For C2 and C3, EC decreased by 16% and 27%, respectively, when comparing the results of the first and third simulations. This indicates a decrease in the amount of solutes in the water and, therefore, less and less leaching of contaminants from the material forming the column fill.

Diatomite, as an almost chemically inert material [69] with a neutral and slightly alkaline pH [70], had a practical impact on the results of the experiment in terms of pH. The average pH value in the synthetic stormwater runoff was 6.88. As shown in Table 12, during the first rainfall simulation, the pH in column 1 increased by 17% relative to the model rain, while in C2 and C3, it remained almost unchanged (a 2% decrease relative to the model rain). After the third rainfall simulation, the pH in all columns was close to the pH of the model rain. The largest change in pH between the first and third rains was 15% and occurred for C1. In contrast, for C2 and C3, it was only a 4% change. The aforementioned correlations for C1 may indicate leaching of contaminants from the material filling this column. The range for the average pH values during the experiment was as high as 1.21 for C1, while for C2 and C3, it was 0.30 and 0.29, respectively. The addition of diatomite kept the pH of leachates from C2 and C3 almost constant.

#### 3.2.2. Removal of Nutrients in Columns

**Figure 6:**
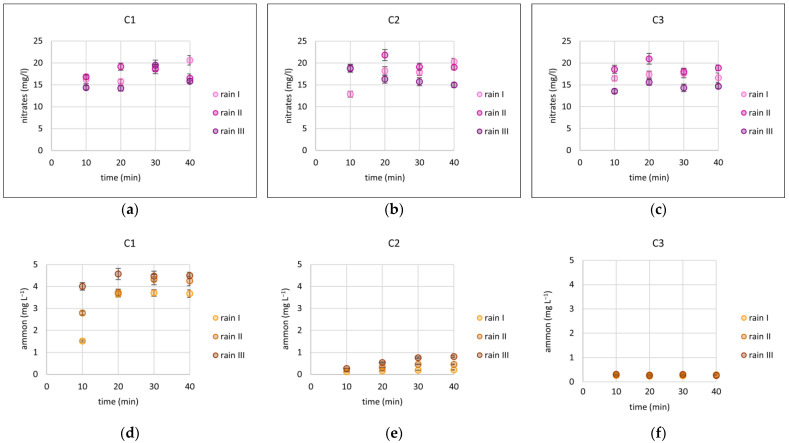

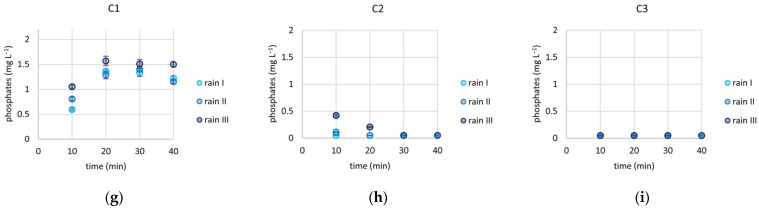
Changes in nitrate (**a**–**c**), ammonium (**d**–**f**), and phosphate (**g**–**i**) concentrations over time for three rainfall simulations (I, II, III) in three columns (C1, C2, C3). Error bars are indicated on the graphs. When the concentration of a given ion was below the detection limit, error bars were not provided.

The magnitudes of the concentrations of individual ions determined during the experiments are illustrated in Figure 6. The ion that showed an increase in concentration during the conducted rainfall simulations of the nitrate ion. On average, relative to the model rain, the increases in concentration were 15%, 19%, and 13% and, thus, the average NO_3_^−^ concentrations were 17.19, 17.82, and 16.89 mg L^−1^ for columns C1, C2, and C3, respectively. On the other hand, another ionic form of nitrogen (i.e., ammonium ion) showed a decrease in concentration regardless of column filling. On average, the concentrations of NH_4_^+^ relative to the model rain decreased by 24%, 92%, and 94%; that is, the average concentrations were 3.77, 0.38, and 0.28 mg L^−1^ for columns C1, C2, and C3, respectively. Diatomite, which was added into C2 and C3, caused a significant change in ammonium ion concentration, which was already registered in a representative sample taken after 10 min. A concentration of 5 mg L^−1^ decreased to 0.12 and 0.27 mg L^−1^ for C2 and C3, respectively. For phosphate, the lowest variability in concentration was observed in C1. The average concentration was 1.23 mg L^−1^, and the range was 0.94 mg L^−1^. For C2 and C3, the changes in concentrations occurring in the first minutes of the experiment reached the level < MQL, where an average value of MQL/2 (i.e., 0.05 mg L^−1^) [71] was used for calculations.

**Figure 7:**
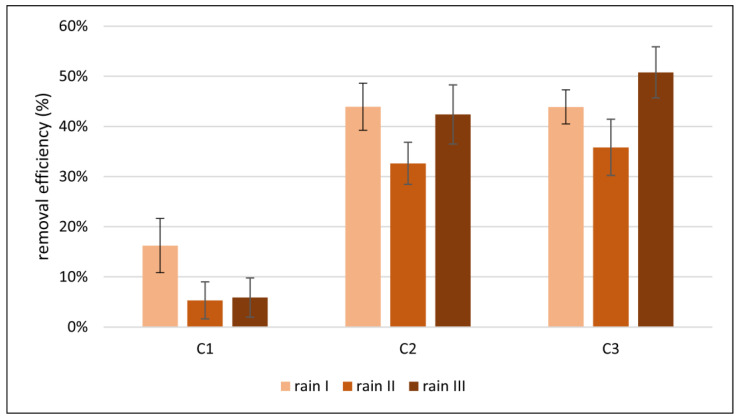
Removal efficiency of nitrogen load (sum of N-NO_3_^−^ and N-NH_4_^+^) for three rainfall simulations (I, II, III) in three columns (C1, C2, C3).

**Figure 8:**
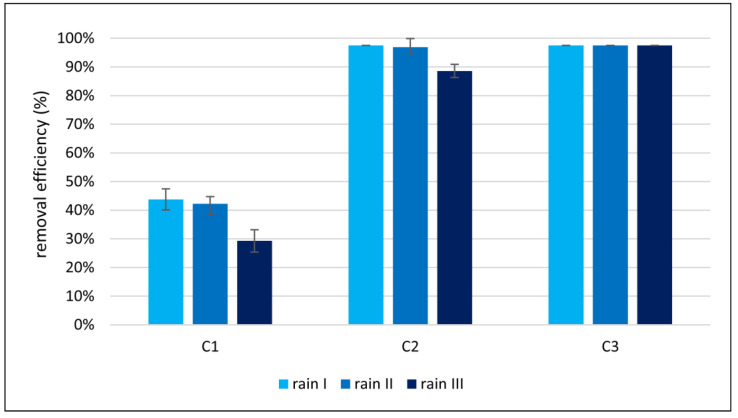
Efficiency of phosphorus load removal for three rainfall simulations (I, II, III) in three columns (C1, C2, C3).

Analyses of nitrogen and phosphorus load removal in the experiments were also performed. The removal of nitrate (the sum of N-NO_3_^−^ and N-NH_4_^+^) and phosphorus P-PO_4_^3−^ was estimated by taking into account the size of the load and, therefore, the amount of water flowing out of each column during the experiments. The results of the conversions are illustrated in Figure 7 and Figure 8.

The removal efficiency of nitrogen (N-NO_3_^−^ and N-NH_4_^+^) in C1 was the lowest with an average value for all experiments of only 9%. The addition of diatomite in C2 definitely improved the nitrogen removal efficiency, which averaged about 40%; therefore, compared to the removal efficiency for C1, it was increased by more than 4 times. Increasing the addition of diatomite in C3 compared to C2 also contributed to a further increase in nitrogen removal; however, the increase was not great at only 10%. The average nitrogen removal increased to about 44%.

It is clear that the leaching of nutrients is largely determined by the amount of precipitation and the concentration of the nutrient load, as well as the reaction of the leaching/adsorption environment and the type of sorption material. In the case of nitrogen compounds, the presence of particular forms of nitrogen in the soil solution is also determined by the presence of living organisms carrying out reactions under anaerobic conditions—in terrestrial ecosystems, biological nitrogen fixation takes place in the presence of nitrogenous bacteria; in aquatic ecosystems, cyanobacteria are responsible for these processes. In the case of the conducted research, the columns did not contain humic compounds or roots of papilionaceous plants. In addition, the simulation of intense rainfall with limited contact time between the medium and sorption material meant that the processes of conversion of various forms of nitrogen occurred in a negligible way. Nevertheless, the addition of diatomite, a common component of deposits used in N-NH_4_^+^ removal [22], as expected, effectively reduced the concentration of ammonium ions. The ammonium ion is susceptible to retention by the sorption complex, while the nitrate ion remaining in soil solution is easily leached and enters groundwater and surface water. As opposed to ammonium nitrogen forms, the oxidized form of nitrogen in the form of N-NO_3_^−^ is a more stable form of mineral nitrogen, but it remains mainly in the soil solution, from which it can be easily reached, as has been proven in ongoing studies.

Phosphorus, like nitrogen, was removed at the lowest rate in C1. For all three rainfall simulations, the average removal efficiency was 38%. It is worth noting that the removal efficiency decreased with subsequent experiments. A comparison of the results for the first and third rainfall simulations showed a decreasing phosphorus removal efficiency of 33%. A similar relationship was observed in C2, but the decrease in phosphorus removal volume was 9%. Nevertheless, the addition of diatomite in the C2 column increased the average phosphorus removal efficiency. For all experiments, it was 96%—almost 2.5 times higher than for C1. For C3, where the addition of diatomite was even greater than in C2, phosphorus was removed most efficiently from the aqueous solution, and the average phosphorus removal efficiency for all three experiments was over 97%.

In the case of phosphorus, due to the lack of a gaseous form, phosphorus does not penetrate the atmosphere, so the biogeochemical cycle of this element occurs between terrestrial and aquatic ecosystems. Phosphorus accumulated in the soil, depending on the nature of its binding (physical or chemical) and the soil/water conditions, can be released in the soil and transformed into phosphorous forms leached deep into the profile or into soil and open water [16]. Soil particles, such as aluminum and iron oxides, clay minerals, and amorphous minerals, which can bind anions, sorb phosphorus in the form of orthophosphates in an amount that depends on the pH and exchange capacity of soil anions, which significantly limits their mobility in the soil/water environment [22,72]. In the case of the experiment conducted where one of the biogenic substances was phosphorus of inorganic origin only, phosphate ions are effectively retained by the various fractions of the columns, (most efficiently on columns enriched with diatomite). As a result, the inorganic phosphorus present does not show significant mobility, is not released into the environment, and is retained in the rain garden.

Based on the removed nitrogen and phosphorus loads, adsorption capacities were also determined for the materials filling each column (Table 13). Regardless of which ions were considered, the smallest adsorption capacity values (averaging 0.5∙10^−3^ and 0.2∙10^−3^ mg g^−1^ for nitrogen and phosphorus, respectively) were obtained for C1. For nitrogen, the average adsorption capacity values calculated for the three model rainfalls for C2 and C3 were 5.4 and 6.3 times higher than for C1, respectively. An increase in nitrogen absorption capacity was achieved with the addition of diatomite into the filling of columns. The higher the proportion of this material was, the higher the removal efficiency. A similar relationship was observed for P-PO_4_^3−^. The mixture of sand and diatomite showed significantly higher adsorption capacity values than sand alone. The addition of diatomite increased the adsorption capacity relative to C1 by 3.0 and 3.4 times in C2 and C3, respectively.

## 4. Discussion

Using two-phase rain gardens, the authors of [73] proved that it is possible to achieve nitrate removal efficiencies of 78–91%. From their results, it was possible to conclude that nitrate removal occurs mainly in the saturated zone, with some additional removal in the unsaturated zone. In another study [74], researchers have also compared the nutrient removal efficiency of a conventional (sub-drainage) rain garden with a two-phase column with simulated drainage, and they also observed significantly higher nitrate removal (63%) in the two-phase rain garden compared to a conventional rain garden (39%). Similar experiments have been conducted by various authors [75,76,77] who proved the beneficial effects of the presence of water saturation conditions on nitrate removal in rain gardens.

For lower initial nitrate concentrations (less than 1.0 mg L^−1^), lower removal efficiency results are obtained, as confirmed in a previous study [14] in which the researchers reported nitrate removal efficiencies of less than 50%.

Zhang et al. [78] have studied the effect of the addition of WTRs on nitrogen removal (TN, N-NH_4_^+^, and N-NO_3_^−^) and showed that the addition of 10% WTRs did not significantly affect N-NH_4_^+^ and N-NO_3_^−^ removal [78].

For nitrate to be removed with high efficiency, there is a need to

- ensure favorable biogeochemical conditions, which are associated with long retention times;
- the presence of a soil environment that facilitates sorption of the negatively charged nitrate ion; and
- the occurrence of denitrification processes.

Xie et al. [41], by using sorption kinetics, exhibited that the sorption capacity decreased from 4.52 mg g^−1^ (pH 5.0) to 1.09 mg g^−1^ (pH 10.0) as a function of pH on raw diatomite. On the other hand, Wajima et al. [42] determined how NH_4_^+^ and PO_4_^3-^ were handled when using paper sludge ash and product-20—a zeolite synthesized from paper sludge ash with the addition of diatomite. The use of diatomite increased the adsorption capacity for the ammonium ion by 5.5 times. In our study, a similar increase in adsorption capacity was obtained in C2 and C3 relative to C1. In contrast, in the study of Wajima et al., the addition of diatomite caused a decrease in the phosphate ion adsorption capacity by as much as 3.7 times; in our study, the change in column filling had the opposite effect. Okada et al. [79] used kaolinite (a silicate) to prepare a KAS composite. In their study, ammonium ions were removed with an efficiency of almost 85%. In contrast, our experiments used diatomite, which is a rock that is rich in silica. The addition of this silica made it possible to achieve a decrease in the concentration of these ions by more than 90%. Another natural mineral used to remove ammonium from solutions is sepiolite, which allowed for the removal of 60% of NH_4_^+^ ions [80]. Balci and Dinçel [80] also removed phosphate ions in the form of HPO_4_^3−^ using sepiolite; however, their efficiency was half that obtained in our experiments. Their study showed that the uptake of ammonium ions was greater than that of phosphate ions. Sepiolite is a calcium carbonate; thus, the mechanism of phosphate ion uptake mainly involves the formation of calcium phosphate. A similar conclusion regarding the removal of phosphate ions was reached by Jha et al. [81], who removed NH_4_^+^ and PO_4_^3−^ using steel-making slag, a by-product that contains mainly CaO, Fe_2_O_3_, and SiO_2_. In addition, according to the authors, the mechanism of NH_4_^+^ uptake includes sorption by the porous silica surface of the samples. Hence, the use of diatomite in our study increased the removal of selected nitrogenous impurities.

A 61.1% removal efficiency value for N-NH_4_^+^ was achieved with natural zeolite at an initial concentration of 100 mg N-NH_4_^+^ L^−1^ by Rožić et al. [82]. A different absorbent material (Modified Converted Slag) was used by Duan et al. [83] to remove ammonium from wastewaters, where the sample was prepared by mixing with aluminum hydroxide. The maximum adsorption capacity of modified slag for NH_4_^+^ was 2.59 mg g^−1^. The same material performed well for the removal of phosphate (PO_4_^3−^), with a maximum adsorption capacity of 1.185 mg g^−1^.

An article reporting that ammonium is absorbed by diatomite has been published by Zhou et. al. [43], who obtained a maximum adsorption capacity of 0.677 mg g^−1^. However, comparing the results is difficult as Zhou et al. conducted a small-scale experiment in which 20 mg L^−1^ N-NH_4_^+^ and 4.0 g diatomite were used [43]. Şahin et al. [84] studied how the addition of 3 g of diatomite to a 500 mL aquarium containing feed proteins would affect ammonium values. When the ammonium values were compared between the diatomite groups and the control group (40.63 mg L^−1^ ammonium), it was found to be 25.27% and 28.62% lower in the D1 and D2 groups, respectively, where D1 is diatomite in powder form and D2 is diatomite with a grain diameter of 1–3 mm [84].

Other researchers [22] have studied the adsorption capacity of diatomite in terms of its suitability for water treatment and wastewater treatment. The initial concentration of ammonium nitrogen ranged from 1.74 to 3.24 mg N-NH_4_^+^ L^−1^, and the experiments measured the changes in ammonium nitrogen in conditioned water on a diatomite bed. The reduction in concentration increased with increasing bed operation time, with the complete removal of N-NH_4_ even being achieved. The diatomite-enriched filter bed was found to be a good carrier of nitrifying bacteria. A similar study on the effectiveness of a diatomite bed in reducing N-NH_4_^+^ concentrations has been conducted by Papciak et al. [85], who reported the maximum efficiencies of beds with two different water flow regimes in the columns to be 97 and 99%.

In this study, the dissolved phosphorus removal efficiency (mass reduction) of columns 2 and 3 was 93.78 and 97.25%, respectively, at a P-PO_4_^3−^ per inlet concentration of 0.59 mg·L^−1^. A similar level of reduction (96.10%) at a dissolved phosphorus concentration half as high (0.28 mg·L^−1^) has been reported by Zhang et al. [78], who used columns containing water treatment residues (WTRs) (volume ratio: 90% garden soil + 10%WTRs) that contain Fe and Al oxides, thus promoting the adsorption of phosphorus. A study of P-PO_4_^3−^ using WTRs at higher concentrations per inlet (1.52 mg·L^−1^) was conducted by Lee et al. [86]. They observed, in a column of 90% sand + 10% WTR, a reduction of 95.5% P-PO_4_^3−^ and, in a column of 85% sand + 10% WTR + 5% compost, a lower reduction of 94.8% P-PO_4_^3−^.

A high (89 to 99%) efficiency of phosphate removal was also obtained [73,74] during studies of simulated urban runoff events. This fact was explained by the sorption of phosphate in the soil substrate in rain gardens. It is worth noting that, due to the potential accumulation of phosphate in the soil substrate, the phosphate removal capacity will decrease over time [74].

Ma et al. [87] studied the Orto-P adsorption capacities of four special filtration media: perlite, zeolite, granular activated carbon (GAC), and engineered media (EM). The examination of these materials revealed the adsorption capacity to range from 0.01 to 7.82 mg g^−1^ [87]. However, it is impossible to compare the results with those obtained in our study, as our study focused on removal not with pure additives but using mixtures with sand (0.50∙10^−3^ to 0.62∙10^−3^ mg g^−1^), where diatomite is only present at rates of 33.3% and 20% by weight.

The results of the nutrient removal studies mentioned above and other studies are presented below in Table 14.

## 5. Conclusions

This study examined and compared the nutrient removal efficiencies in three filter columns using synthetic rainwater. Pollutant removal efficiencies (NO_3_^−^, NH_4_^+^, and PO_4_^3−^) for each column while operating during rainfall for a duration of 40 min were evaluated using three runoff simulations that reflected rainfall occurring in Polish cities. Key results were as follows:

- In all columns, the concentration (the Event Mean Concentration (EMC)) of nitrate increased compared to the initial concentration. In column C1, it increased by an average of 11%; in column C2, it increased by 22%; and in column C3, it increased by 15%.
- Column I, which was the column without the addition of diatomite, showed the weakest removal efficiency of nutrients. The concentration of ammonium ions decreased by 27% on average compared to the initial concentration. For phosphate, there was a 38% decrease in concentration.
- Column I was the least effective in removing selected contaminants as a standard container rain garden. It was shown that the addition of diatomite was more effective in retaining selected contaminants.
- Column II, which was the column in which sand and diatomite were mixed in a weight ratio of 4:1, respectively, removed phosphates with 94% efficiency. The addition of diatomite made column II more effective in retaining ammonium compared to C1 (the concentration reduction was 93%).
- Column III, which was prepared at a weight ratio of 2:1 (sand:diatomite) was most effective in removing NH_4_^+^ and PO_4_^3−^. Compared to C2, the ammonium removal efficiency increased to 94% while the phosphate removal efficiency increased to 98%.

When designing rain gardens to purify the pollutants analyzed in this article, practitioners should use certain sorbents, such as tested diatomite. Further work is needed to confirm these findings for the treatment of other pollutants.

The described study is only a prelude to extensive experiments planned in the future for the design and construction of rain gardens, where the primary sorbent will be diatomite. The observed variations in the concentrations of the analyzed ions in the outflow from the gardens—with higher removal efficiencies during periods of lower inflow intensity—also indicate the direction of analysis in terms of efficiency during rainfall periods with respect to other parameters. Another parameter that will also be the focus of further research is the effect of initial concentration on the effectiveness of their reduction. Modification of the garden’s construction approach is also planned; in this study, a garden without vegetation based on materials characterized by high water permeability was analyzed, while further studies will also consider modifications to its construction. In addition, the authors also plan to perform microbiological analyses (determination of Acidibacter, Flavobacterium, Nocardioides, Pseudomonas, and Sphingomonas) in order to better interpret nutrient removal mechanisms in rain gardens.

The conducted study and the obtained results are of importance to both urban stormwater managers and property owners who, through appropriate education and information campaigns, can expect to receive or develop revised rules for the construction of rain gardens capable of effectively removing the biogenic pollutants present in urban stormwater runoff.

## Figures and Tables

**Table 1:** Filtration coefficients (k_10_) and porosity (n) for the various materials, along with the nature of their permeability.

Material	k_10_ m s^−1^	Nature of Permeability [49]	n
Sand	6.4∙10^−4^	Good	0.33
Gravel	1.2∙10^−2^	very good	0.33
Dolomite	7.2∙10^−3^	very good	0.45
Diatomite	9.4∙10^−3^	very good	0.60
Sand + Diatomite C2	7.5∙10^−4^	Good	0.38
Sand + Diatomite C3	7.1∙10^−4^	Good	0.39

**Table 2:** Results of granulometric analyses of materials used in column construction.

Material	Si %	Sand Sa %			Gravel Gr %			d_10_ mm	C_u_	C_c_	Classification
		fSa %	mSa %	cSa %	fGr %	mGr %	cGr %				
Sand	-	3	62	35	-	-	-	0.31	1.8	0.9	Medium sand
Gravel	-	1	1	16	57	25	-	1.30	3.6	1.3	Fine gravel
Dolomite	-	-	-	2	48	50	-	2.60	2.8	0.9	Medium gravel
Diatomite	-	-	2	95	3	-	-	0.90	1.3	1.1	Coarse sand
Sand + Diatomite C2	-	1	50	48	1	-	-	0.32	2.2	0.8	Medium sand
Sand + Diatomite C3	-	1	47	50	2	-	-	0.33	2.6	0.7	Coarse Sand

Si—silt fraction (>0.002 to ≤0.063 mm), Sa—sand fraction (>0.063 to ≤2.0 mm), fSa—fine sand fraction (>0.063 to ≤0.2 mm), mSa—medium sand fraction (>0.2 to ≤0.63 mm), cSa—coarse sand fraction (>0.63 to ≤2.0 mm), Gr—gravel fraction (>2.0 to ≤63mm), fGr—fine gravel fraction (>2.0 to ≤6.3 mm), mGr—medium gravel fraction (>6.3 to ≤20 mm), cGr—coarse gravel fraction (>20 to ≤63 mm), d_10_—effective size mm, C_u_—coefficient of uniformity, $C_{u}=\frac{d_{60}}{d_{10}}$, C_c_—coefficient of curvature, and $C_{c}=\frac{{\left(d_{30}\right)}^{2}}{\left(d_{10}\cdot d_{60}\right)}$, where d_60_, d_30_, and d_10_ are the particle diameters corresponding to 60%, 30%, and 10% fineness values on the cumulative particle size distribution curve, respectively.

**Table 3:** Stages of degassing process.

Target Temperature °C	Heating Speed ° min^−1^	Degassing Time min
80	2	30
120	2	30
350	5	400

**Table 4:** Laboratory multifunction instrument CX-505.

Parameter	pH	EC	KCl	NaCl	O_2_
Range of determination	−6.000–20.000	0–1999.9 mS cm^−1^	0–239 g L^−1^	0–296 g L^−1^	0–60 mg L^−1^

**Table 5:** Reported pollutant concentrations in rainwater in Poland [59] and in tributaries to stormwater BMPs [60].

	N-NO_3_^−^ and NO_2_^−^ mg L^−1^	N-NO_3_^−^ mg L^−1^	NO_3_^−^ mg L^−1^	N-NH_4_^+^ mg L^−1^	NH_4_^+^ mg L^−1^	TP mg L^−1^	PO_4_^3−^ mg L^−1^
Max concentration in rainwater (GIOS)	2.99		13.24	3.78	4.86	0.58	1.78
Max concentration in tributary BMP DBase		28.00	124.00	2.19	2.82		6.98
Median concentration in tributary BMP DBase		0.44	1.95	0.29	0.37		1.37

**Table 6:** Synthetic stormwater runoff.

Biogenic Component	Source	Concentration mg L^−1^
NH_4_^+^	NH_4_Cl	5.0
NO_3_^−^	KNO_3_	15.0
PO_4_^3−^	Na_2_HPO_4_	2.0

**Table 7:** Estimation of the amount of stormwater supplying the column/rain garden.

No.	Time Interval min	Precipitation Amount mm	Amount of Stormwater Flowing into the Column/Garden dm^3^
1	5	6.73	1.98
2	5	12.74	3.75
3	5	5.13	1.51
4	5	2.68	0.79
5	5	2.28	0.67
6	5	2.00	0.59
Total	30	31.56	9.30

**Table 8:** Oxide analysis for different raw and calcined diatomite fractions.

	Oxide Composition wt.%							
	SiO_2_	Al_2_O_3_	Fe_2_O_3_	K_2_O	SO_3_	TiO_2_	CaO	Others
Raw diatomite	77.76	13.52	4.86	2.31	0.43	0.55	0.40	0.17

**Table 9:** Identified phases in raw diatomite.

	Raw Diatomite			
Identified Phase	Quartz	Illite-2M	Kaolinite	Albite
Chemical Formula	SiO_2_	(K, H_3_O)Al_2_Si_3_AlO_10_(OH)_2_	Al_2_(Si_2_O_5_(OH)_4_)	NaAlSi_3_O_8_
Amount of Phase %	22.2	29.2	30.7	17.9

**Table 10:** Porous texture parameters of diatomite.

	Porous Texture Parameters						
Sample	SBET m^2^ g^−1^	$V_{tot}^{0.99}$ cm^3^ g^−1^	$V_{mik}^{DR}$ cm^3^ g^−1^	$V_{mez}^{BJH}$ cm^3^ g^−1^	$S_{mik}^{DR}$ m^2^ g^−1^	$S_{mez}^{BJH}$ m^2^ g^−1^	D_A_ nm
Raw diatomite	29.003	0.0559	0.012	0.053	33.876	24.752	7.708

**Table 11:** Average EC values for individual columns for three experimental rainfalls (rain I–III).

		C1	C2	C3
		EC µS cm^−1^	EC µS cm^−1^	EC µS cm^−1^
Rain	I	121.73	129.58	147.60
	II	127.75	119.35	133.15
	III	128.20	108.80	107.75

**Table 12:** Average pH values for individual columns for three experimental rainfalls (rain I–III).

		C1	C2	C3
		pH	pH	pH
Rain	I	8.05	6.76	6.72
	II	7.50	6.81	6.70
	III	6.84	6.51	6.43

**Table 13:** Adsorption capacity (mg g^−1^) for sand and mixtures of sand and diatomite.

		Removal in the Column Layer mg g^−1^	
Column	Rain	Nitrogen (sum N-NO_3_^−^ and N-NH_4_^+^)	Phosphorus P-PO_4_^3−^
	I	0.9∙10^−3^	0.21∙10^−3^
C1	II	0.3∙10^−3^	0.21∙10^−3^
	III	0.3∙10^−3^	0.13∙10^−3^
	I	2.9∙10^−3^	0.59∙10^−3^
C2	II	2.1∙10^−3^	0.57∙10^−3^
	III	2.7∙10^−3^	0.50∙10^−3^
	I	3.1∙10^−3^	0.62∙10^−3^
C3	II	2.5∙10^−3^	0.62∙10^−3^
	III	3.5∙10^−3^	0.60∙10^−3^

**Table 14:** Uptake of nitrate NO_3_^−^, nitrogen N-NO_3_^−^, ammonium NH_4_^+^, nitrogen N-NH_4_^+^, phosphate PO_4_^3−^, and phosphorus P-PO_4_^3−^ by zeolites and other sorbents.

Nutrients	Adsorbent Type (Name, Type, Description)	Adsorption Capacity mg g^−1^ or Efficiency of Removal %	Initial Concentration mg L^−1^	References
N-NO_3_^−^	fine gravel (3.2–12.7 mm diam.; 0.15 m deep), soil medium—mixture of sand, topsoil, and compost in a 6:2:2 volume ratio (0.85 m deep), and fine gravel (of 0.15 m depth and soil medium of 0.35 m depth)	78–91%	20.0	[73]
	clay, silt, and sand are 8.80, 79.9, and 11.31%	49.84%	0.87	[14]
	mixture of sand, topsoil, and compost (6:2:2, volume ratio), soil medium consists of 90.6% sand, 6.9% silt, and 2.5% clay with 0.7% organic matter	42–63 and 29–39%	5.0–50.0	[74]
	column types: GS (97% garden soil + 3% wood chips, submerged zone 300 mm) GWS (87% garden soil + 10% WTRs + 3% wood chips, submerged zone 300 mm)	GS = 92.37 ± 3.89% GWS = 93.96 ± 2.82%	3.25	[78]
NH_4_^+^	paper sludge ash	0.2 mg g^−1^	10.0	[42]
	product-0 synthesized without diatomite addition	0.5 mg g^−1^	10.0	[42]
	product-20 synthesized from solution with initial Si concentration of 20 g L^−1^	1.1 mg g^−1^	10.0	[42]
	converter slag modified by aluminum hydroxide	2.59 mg g^−1^	10.0–50.0	[83]
	γ-alumina/potassium aluminosilicate gel (KAS)	10.6 mg g^−1^ (84.8%)	36.1	[79]
	sepiolite	63.0 mg g^−1^ (60%)	150.0–4000.0	[80]
	steel-making slag	5.42%	180.0	[81]
N-NH_4_^+^	diatomite (2–4 mm)	0.67 mg g^−1^	20.0	[43]
	natural zeolite (0.2–0.5mm)	61.1%	100.0	[82]
	D1 diatomite (powder) D2 diatomite (1–3 mm)	25.27% (D1) 28.62% (D2)	-	[84]
	diatomite 0.75–1.02 mm	Dependent on the operating time of the bed, even 100%	1.74–3.24	[22]
	diatomite 1–3 mm filter bed: height: 1.2 m, • diameter: 35 mm, • granulation: 1–3 mm	97 and 99%	1.60–3.50	[85]
	column types: GS (97% garden soil+3% wood chips, submerged zone 300 mm) GW (90% garden soil + 10% WTRs) GWS (87% garden soil + 10% WTRs+3% wood chips, submerged zone 300 mm)	GS = 62.51 ± 9.03% GW = 90.58 ± 3.95% GWS = 54.28 ± 7.98%	6.63	[78]
PO_4_^3−^	paper sludge ash	2.2 mg g^−1^	10.0	[42]
	product-0 synthesized without diatomite addition	2.0 mg g^−1^	10.0	[42]
	product-20 synthesized from solution with initial Si concentration of 20 g L^−1^	0.6 mg g^−1^	10.0	[42]
	raw diatomite	4.52–1.09 mg g^−1^ as a function of pH	50.0	[41]
	converter slag modified by aluminum hydroxide	1.19 mg g^−1^	1.0–30.0	[83]
	steel-making slag	53.2%	950.0	[81]
	surfactant-modified clay	38.50 mg g^−1^	-	[88]
	paper sludge ash	2.2 mg g^−1^	10.0	[42]
P-PO_4_^3−^	column 2 (90% sand + 10% WTR) 95.5 ± 1.9% column 3 (85% sand + 10% WTR+ 5% compost) 94.8 ± 2.6%	85–95.5%	1.52	[86]
	fine gravel (3.2–12.7 mm diam.; 0.15 m deep), soil medium—mixture of sand, topsoil, and compost in a 6:2:2 volume ratio (0.85 m deep), fine gravel (of 0.15 m depth and soil medium of 0.35 m depth)	94–99%	10.0	[73]
DP	column types: GS (97% garden soil +3% wood chips, submerged zone 300 mm) GW (90% garden soil + 10% WTRs) GWS (87% garden soil + 10% WTRs + 3% wood chips, submerged zone 300 mm)	GS = 87.25 ± 4.88% DP GW = 96.10 ± 2.84% DP GWS = 93.73 ± 2.76% DP	DP = 0.28	[78]
Orto-P	perlite	0.01 mg g^−1^	0.5	[87]
	zeolite	0.13 mg g^−1^	0.5	[87]
	granular activated carbon (GAC)	1.16 mg g^−1^	0.5	[87]
	EM-packed HFCs	7.82 mg g^−1^	0.5	[87]
HPO_4_^2−^	γ-alumina/potassium aluminosilicate gel	12.7 mg g^−1^ (48.6%)	96.0	[79]

## Data Availability

The datasets will be made available by the corresponding author upon reasonable request.

## Supplementary Materials

The following supporting information can be downloaded at: https://www.mdpi.com/article/10.3390/ma17246279/s1, Figure S1: Diagram of a device used to determine the permeability coefficient; Table S1: Materials parameters used for porosity calculations.
