# Supplementary material for: Effect of Diatomite Application on the Removal of Biogenic Pollutants in Rain Gardens
Source: Materials (Basel). 2024 Dec 22;17(24):6279. doi: 10.3390/ma17246279 (PMC11727856; doi:10.3390/ma17246279)
Supplement: Supplementary file 1 [file materials-17-06279-s001.zip › materials-3353164-supplementary.pdf]

Supplement to subsection *2.1 Material for construction of columns – rain gardens*

Notes on Table 1

The filtration coefficient due to the fact that all materials used to construct the column were of good or very good permeability and were tested in the ZWK II device produced by ZAN (Cracow, Poland)**.** This device is used to determine the water permeability of coarse soil (sand and gravel). The principle of operation is shown in the simplified diagram below. Water can flow through the soil sample both from below (as in the diagram) and from above. A constant hydraulic gradient is maintained during the test. Calculations are carried out in accordance with Darcy's low for laminar flow:

$$k = \frac{Q \cdot l}{A \cdot \Delta h}$$

where:
k – coefficient of permeability, m s^{-1}
Q – flow rate, m^3 s^{-1}
l – height of specimen, m
A – cross-sectional area of specimen, m^2
Δh – difference in head of water, m

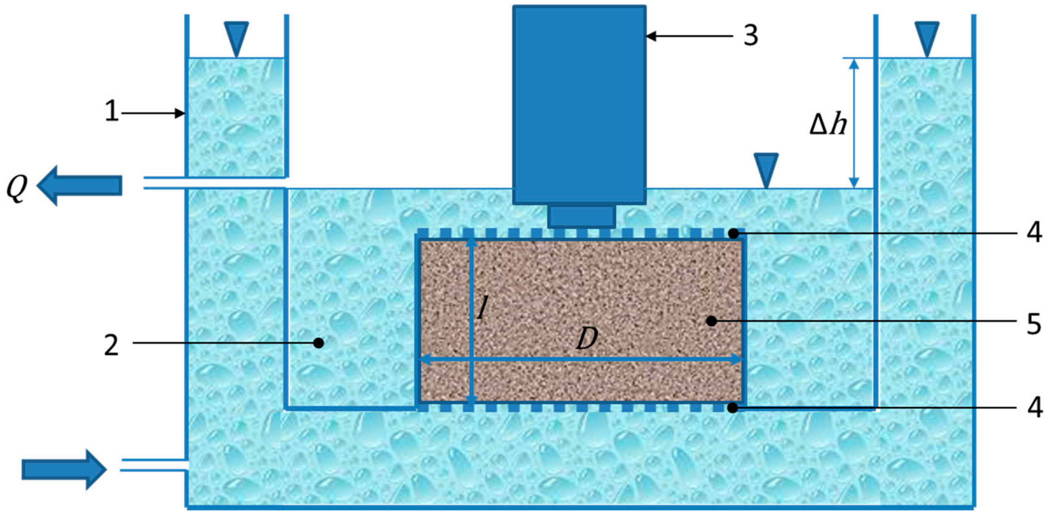

Figure S1. Diagram of a device used to determine the permeability coefficient.

Key
1 – external reservoir
2 – internal reservoir
3 – vertical weight
4 – perforated base plated
5 – specimen (*D*- diameter)

Porosity of the materials used to construct the column is high, the grain size is uniform, the materials in the column have been placed in layers by piling dry substances with little vibratory compaction. The water velocity through the columns also indicates high porosity. Porosity was determined by the formula:

$$n = \frac{\rho_d - \rho_s}{\rho_s}$$

where:
n – porosity, -
ρ_d – dry density, g m^{-3}
ρ_s – particle density, g m^{-3}

The volumetric density of the soil skeleton (**dry density**) was determined by the formula:

$$\rho_d = \frac{\rho}{1 + w}$$

where:
ρ_d - dry density, g m^{-3}
ρ – bulk density, g m^{-3}
w – water content, -

Bulk density was determined in accordance with PKN-CEN ISO/TS 17892-2 by the direct linear measurment method. The principle of the method is to weigh a specimen of known volume.

Water content was determined in accordance with PKN-CEN ISO/TS 17892-1. Samples were dried in an SML drier (ZALMED, Warsaw, Poland) at a constant temperature of 105°C ± 5°C and weighed with an accuracy of 0.01g.

Specific density of the skeleton was determined in a helium pycnometer for diatomite (Pycnomatic ATC, Thermo Fisher Scientific, Massachusetts, USA), and for the other materials in a water pycnometer according to PKN-CEN ISO/TS 1789-3.

The results are shown in the table S1.

Table S1. Materials parameters used for porosity calculations.

**Material**	ϱ g m^{-3}	w	ϱ_s g m^{-3}	n
Sand	1.77	0.003	2.65	0.33
Gravel	1.79	0.005	2.65	0.33
Dolomite	1.56	0.01	2.81	0.45
Diatomite	0.89	0.01	2.24	0.60
Sand + Diatomite C2	1.6	0.007	2.57	0.38
Sand + Diatomite C3	1.55	0.008	2.51	0.39
